# rbpTransformer: A novel deep learning model for prediction of piRNA and mRNA bindings

**DOI:** 10.1371/journal.pone.0324462

**Published:** 2025-06-25

**Authors:** Ahmet Gürhanlı

**Affiliations:** Department of Computer Engineering (English), Istanbul Topkapi University, Istanbul, Türkiye; National Institute of Child Health and Human Development (NICHD), NIH, UNITED STATES OF AMERICA

## Abstract

An important issue in biotechnology is predicting whether a piRNA and an mRNA will or will not bind. Research and treatment of diseases, drug discovery, and the silencing and regulation of genes, transposons, and genomic stability may all benefit from accurate binding predictions. The literature offers numerous deep-learning models for piRNA and mRNA binding prediction. However, a proper adjustment of the effective transformer model and the impact of important design alternatives has not been evaluated thoroughly. This paper summarizes the models available in the literature, briefly introduces transformers, then offers a novel deep learning model and evaluates various design alternatives, including k-mer size, number of core modules, choice of optimization algorithm, and whether to use self-attention. The results show that rbpTransformer can be a good candidate for building deep AI models to predict the binding of piRNA and mRNA sequences with an AUC value of 94.38%. The test results also reveal how the design affects the model’s accuracy.

## Introduction

RNA molecules are crucial in understanding cellular processes, genetic information transfer, and many other key issues that will assist studies about drug discovery and disease mechanisms. piRNA and mRNA interactions and RNA binding processes must be understood and predicted accurately to support medical research and development efforts. Artificial intelligence-based computational methods have become very effective in RNA binding prediction after deep learning has been widely adopted and solved accuracy and efficiency problems.

In previous research, several artificial intelligence-based and computational methods have been developed for predicting piRNA-mRNA bindings and, a related problem, RNA-protein bindings.

Several tools have been developed to predict the bindings between piRNA and mRNA sequences. High-throughput sequencing has been utilized to assess piRNA and mRNA expression levels to detect possible interactions. Applications like RNAfold or Mfold are developed to forecast RNA’s secondary configurations, which can potentially affect binding interactions. Some tools work by comparing sequences, revealing shared regions between piRNAs and mRNAs, and indicating possible interaction sites. An accurate deep learning model can be very helpful in developing new tools with higher prediction accuracy.

Many machine learning algorithms have been proposed for piRNA-mRNA interactions based on sequence features and biological context. Sequence patterns, structural characteristics, and thermodynamic stability have been employed in training algorithms to predict interactions.

Support-vector machine-based methods used sequence and structural features derived from RNA to classify RNA-binding proteins. Random forests and decision trees have been proposed for handling complex RNA data. Convolutional neural networks and recurrent neural networks have been proposed to figure out the representation of RNA sequences. Sequence-based approaches focused on finding similarities between RNA binding sites. On the other hand, structure-based techniques tried to predict secondary structures to model binding sites. Deep learning models such as graph neural networks and transformers used the power of GPU processing and obtained very good accuracy values. Finally, some statistical and probabilistic methods have been used to model binding motifs and dependencies between features.

Short, continuous nucleotide sequences of length k that are derived from a longer RNA sequence are known as k-mers. These k-mers are fundamental units or characteristics that aid in capturing local sequence patterns and motifs that may affect binding affinity and specificity in RNA sequence binding prediction.

Recently, advances in GPU processing have made deep learning the leading choice in RNA binding classification. This research contributes to the literature by offering a new transformer-based model, evaluating k-mer subsequences in forward and backward directions, empirically evaluating various leading backpropagation algorithms, and evaluating the optimal core number with various k-mer sizes. The following section provides a summary of recent research in the field.

## Related work

Since the problem is critical for improving medical studies, many research works have been carried out recently [1]. This section will provide a summary of these research papers.

The piRNAdetect [2] technique is designed to accurately predict piRNAs in genome sequences using computational methods. The approach involves categorizing piRNA sequences with similar motifs in a training dataset and extracting predictive features using n-gram models (NGMs). These NGM-based features are then utilized to create a support vector machine for precise identification of new piRNAs.

Yang and his team [3] developed a deep learning framework that utilizes multi-head attention to detect piRNA binding sites on mRNA. Within their framework, they initially encode the piRNA and mRNA sequences using one-hot encoding and subsequently apply a combination of convolution and squeezing extraction to reveal distinctive motif patterns. Following this, a multi-head attention sub-network is utilized to extract the concealed rules governing piRNA binding, which emulate the biological process of piRNA target recognition. Lastly, a deep fully connected sub-network is employed to pinpoint the genuine piRNA–mRNA binding pairs.

Yuan and colleagues offered a method for predicting piRNA targets on mouse mRNAs [4]. They developed a support vector machine classifier using a blend of Miwi CLIP-Seq-derived characteristics and position-based features to forecast the potential interactions between piRNAs and mRNAs in mice.

RNA-protein binding is a closely related problem to piRNA and mRNA binding prediction. Instead of focusing on interactions between nucleotide sequences, the researchers focus on interactions between nucleotide and amino-acid sequences and structural analysis of proteins.

Mohibi [5]examined the existing information on RNA-binding proteins and their involvement in the development of tumors, and the possibility of targeting RNA-binding proteins for cancer treatment. The main difficulties associated with analyzing computational CLIP (cross-linking and immunoprecipitation) data were summarized by another work by Hafner [6] and colleagues. They also discussed methods for addressing different sources of background information and identified regions related to binding processes. Additionally, they outlined different applied works of CLIP and the accessible data. Hafner et al. also explored the potential integration of CLIP data with supporting approaches to obtain a comprehensive understanding of RNP (ribonucleoprotein) formation and remodeling. They investigated the movement and timing of RNPs in particular types of cells and within specific parts of cells. They also studied how problems with RNPs can play a role in causing diseases.

Multiple research teams have assembled databases containing pairs that bind in protein-nucleic acid interactions.[7–14].

Alipanahi and colleagues developed a deep learning technique called DeepBind [15], utilizing convolutional neural networks. This method can uncover new patterns in sequences, even when the exact locations of these patterns are unknown. Traditional neural networks typically demand massive training data to accomplish this task.

Pan and Shen introduced a new framework called iDeep [16], which is based on deep learning. They used a combination of a CNN and a deep belief network to figure out the interaction fields and patterns of RNA-binding proteins (RBPs). Their approach involves converting the initial data into a feature space with high-level abstraction that employs many learning stages. This allows for the integration of shared representations across various domains.

deepRKE [17] has been introduced as a comprehensive deep-learning framework for forecasting RNA-protein binding sites. This approach utilizes a two-layer neural network without supervision to acquire the distributed representation of k-mers by taking into account their surrounding context. In comparison to traditional k-mer methods, distributed representations are expected to more effectively identify the underlying relationships and similarities among k-mers.

CRIP [18] utilizes a hierarchical encoding approach (a method that represents data by breaking it into multiple levels, capturing both fine-grained details and broader, abstract structures within the information) based on codons and a hybrid deep learning architecture. This model employs a convolutional neural network (CNN) to extract high-level abstract features, while a recurrent neural network (RNN) captures long dependencies within the sequences.

HOCNNLB [19] stands for “Higher-Order Nucleotide Encoding Convolutional Neural Network-Based Method.” This method is employed for predicting the locations where RNA-binding proteins (RBPs) bind to long non-coding RNA (lncRNA) chains. HOCNNLB employs a sophisticated encoding method, known as high-order one-hot encoding, to represent lncRNA sequences. This encoding takes into account the relationship between nucleotides. Subsequently, the encoded lncRNA sequences are fed into a convolutional neural network (CNN) to forecast the binding sites of RBPs.

GraphProt [20] was presented as a computational framework designed to analyze high-throughput experimental data and learn the binding preferences of RNA-binding proteins (RBPs) using sequence and structure data together. Computational binding models are vital in forecasting RBP binding sites and affinities across all tissue types.

iDeepV [21] is a deep learning technique specifically developed for acquiring distributed representations of RNA sequences. The process starts by utilizing an unsupervised shallow two-layer neural network to autonomously learn the distributed representation of k-mers while taking into consideration their surrounding context. This updated approach captures the underlying association of k-mers by factoring in their similarities. Afterwards, the distributed representations of the input sequences are employed as inputs for a convolutional neural network (CNN) to differentiate between RBP-bound sites and unbound sites.

iDeepE [22] is a computational technique that uses both global and local convolutional neural networks to forecast RNA-protein binding sites based on RNA sequences. The global CNN involves padding RNA sequences to equal lengths, while the local CNN involves splitting the sequences into several overlapping fixed-length subsequences. Deep CNNs are trained for both the subsequences and padded sequences to recognize advanced features, and the outputs from the local and global CNNs are combined to improve the prediction.

DeCBAN [23] method utilizes advanced deep learning techniques to identify interactions between circular RNAs (circRNAs) and RNA-binding proteins (RBPs). It leverages a unique approach that combines dual embeddings to represent RNA sequences and a cross-branch attention neural network for accurate classification. The dual embeddings consist of pre-trained vectors for RNA segments and their associated amino acids, allowing for a comprehensive representation of RNA sequence data. Additionally, the cross-branch attention network addresses the challenge of processing long sequences by incorporating features of different scales and highlighting important information.

PASSION [24] is a type of AI that combines artificial neural network (ANN) and hybrid deep neural network frameworks. The ANN uses the best feature subset for each RBP, selected from six different feature encoding schemes using incremental feature selection and the XGBoost algorithm. At the same time, the stacked codon-based approach is used as input for the hybrid deep neural network.

RDense [25] is a deep learning model designed to forecast interactions between proteins and RNA. This model takes into account RNA sequences, secondary structure data, and a pairwise probability feature derived from RNA secondary structure. It utilizes bidirectional long and short-term memory neural networks (Bi-LSTM) along with densely connected convolutional neural networks (DenseNet) to understand the preferences for protein-RNA binding. The reported average AUC of this model is 0.804. Bidirectional LSTM networks is a type of recurrent neural network that can process sequences both forward and backward. They improve performance on tasks like language modeling and sequence prediction by using LSTM cells to capture long-term dependencies while also taking future context into account.

The next section summarizes the transformer architecture, and the following section introduces the adopted deep learning model for predicting the bindings between piRNA and mRNA sequences.

## Background information for transformers

The famous paper “Attention Is All You Need” [26] introduced transformers. In this section, important parts and equations of the transformer model are revisited as background.

Transformers have two parts: the encoder and the decoder. The encoder part takes the input sequence, and the decoder takes in the shifted output sequence. The model tries to predict the next output based on the input sequence and already generated outputs.

Encoder and decoder inputs are first passed through positional encoding, which injects information about the relative and absolute positions of the input tokens. Sine and cosine functions generate the encoding.


$$\begin{array}{c} {PE}_{\left(pos,2i\right)}=\sin\left(\frac{pos}{10000^{\frac{2i}{d_{model}}}}\right) \end{array}$$ (1)

$$\begin{array}{c} {PE}_{\left(pos,2i+1\right)}=\cos\left(\frac{pos}{10000^{\frac{2i}{d_{model}}}}\right) \end{array}$$ (2)

In equations (1) and (2), “pos” denotes the position, “i” represents the dimension, and “d_model_” is the dimension of output tokens. Each dimension of the positional encoding corresponds to a sinusoidal function. The wavelengths create a geometric progression ranging from 2π to 10000· 2π.

The next process is masking the positionally encoded data. In language modeling or machine translation processes, masking is essential to prevent the model from gaining an unfair advantage by peeking ahead at future tokens during training. There are two main types of masking utilized in transformer models.

In padding masking, the non-uniformity of the input data is addressed. The shorter sequence is padded with dummy data to make both inputs compatible, mostly with -inf (the smallest value).

In tasks like language modeling, the AI model should not see the tokens in future positions relative to the current token. Look-ahead masking replaces future tokens with -inf to make the translation work properly.

Masked sequences are fed into multi-head attention layers. These layers are parallel combinations of scaled dot-product attention modules. The equation that describes the scaled dot-product attention is given below.


$$\begin{array}{c} Attention(Q,K,V)=Softmax\left(\frac{QK^{T}}{\sqrt{d_{k}}}\right)V\; \end{array}$$ (3)

Equation (3) involves working with queries and keys of size d_k_, and values of size d_v_. The process includes calculating the dot products of the query with all keys, dividing each by the square root of d_k_, and then using a SoftMax function to determine the weights on the values. In practical applications, the attention function is computed on a set of queries organized into a matrix Q. Similarly, the keys and values are organized into matrices K and V.

The next section introduces the adjustments to this base transformer model that this research offers to make better binding predictions between RNA sequences.

## Proposed rbpTransformer model

**Fig 1:**
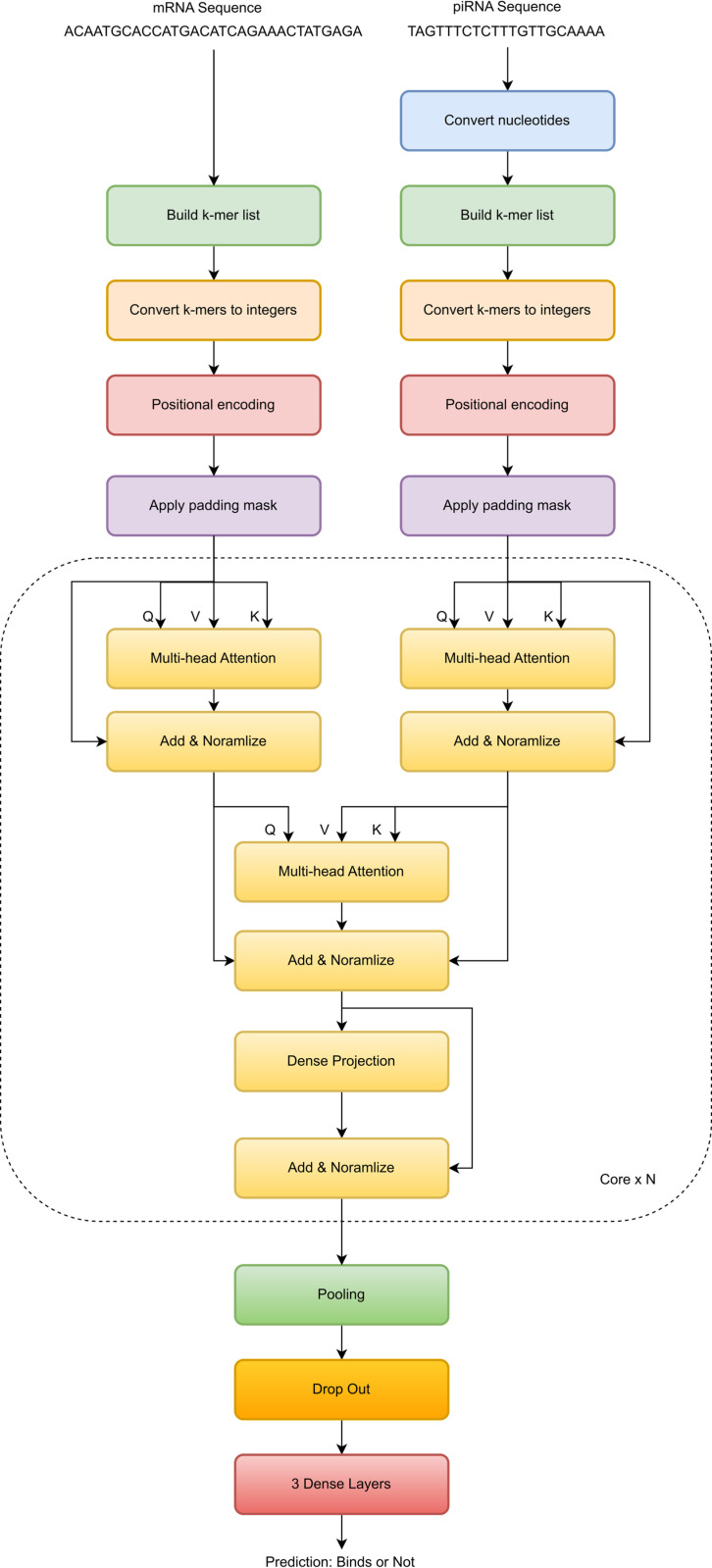
rbpTransformer model. The core part applies both self-attention and cross-attention. The core module can be cascaded to get better accuracy.

The model proposed in this paper is rbpTransformer, which stands for RNA Binding Prediction Transformer. It is illustrated in Fig 1. It takes a piRNA and an mRNA sequence as inputs and makes a binary prediction about their binding relations.

### Conversion of one of the inputs

The model first converts one of the input sequences to the opposite sequence to reflect the bindings between Adenine-Thymine pairs and Guanine-Cytosine pairs. For example, if we take an RNA sequence like TAGTTTCT, it becomes ATCAAAGA after this transformation.

This transformation aims to mark the k-mers with a high potential to bind each other with the same integer. For example, a k-mer such as ACG will likely bind to TGC. If we do not do the conversion, these two k-mers will be represented with different integers, and it might be harder for the AI model to determine the binding relation between them. However, after the conversion, both k-mers will be represented with the same integer, and catching the binding relation will be easier for the deep-learning model.

### Building the k-mer list

The next step is building the k-mer list for each input and representing these k-mers by integers. K-mers are subsequences of length k inside an RNA nucleotide sequence. In this study, high-order k-mers also cover low-order ones. If k is selected as 3, the model will work with k-mers of sizes 1, 2, and 3. Table 1 illustrates the total number of k-mers that will be produced from an imaginary sequence GCATTACG.

**Table 1:** K-mer list for an imaginary nucleotide sequence.

k	k-mers
**1**	G, C, A, T, T, A, C, G
**2**	GC, CA, AT, TT, TA, AC, CG
**3**	GCA, CAT, ATT, TTA, ACG

The table is generated for the imaginary sequence **GCATTACG** and for the maximum k-mer size 3.

A dictionary that covers all possible nucleotide sequences up to the maximum k-mer size is generated. This dictionary maps each possible k-mer sequence to a unique integer. Later, the k-mers in a specific sample are encoded using this dictionary.

### Positional encoding and masking

The positional encoding layer applies the same transformation with the transformers built for natural language processing applications. Just like an NLP transformer encodes the positional relations among words of a sentence, in this application, the same layer represents the positional information of the k-mers in a nucleotide sequence.

The padding mask is necessary because one of the sequences can be shorter than the other. If this is the case, the empty token positions must be filled with the least integer to make the model work properly.

However, the look-ahead mask is not needed in RNA binding applications. NLP transformers take outputs and feed them back to one of the inputs to mask future token positions. This way, wrong interpretations of the input data are avoided. But in our case, we need to interpret both nucleotide sequences as a whole, and the look-ahead mask may degrade the learning performance. Therefore, it is not employed in our model.

### The core

The core part of the model includes 3 multi-head attention modules, 1 dense projection module, and 4 add-and-normalize modules, which are used after each step.

The first two multi-head attention modules are used for self-attention inside a nucleotide sequence. Self-attention is crucial in NLP (Natural Language Processing) applications because it helps the model give meaning to the sentence, and many intricate relationships among words in a sentence are resolved by self-attention. It may still be important in RNA binding prediction because when some sub-nucleotides take place in a specific order, the binding mechanisms may be affected. So, in the baseline model, self-attention is included, but during empirical refinement, a model that does not employ self-attention will be introduced, and a comparison between the two choices will be given.

After self-attention, we add the inputs of multi-head attention components to the output because we do not want to lose critical information available before applying the attention process. Then, the data is normalized to keep it in a certain range. This addition of inputs and outputs and the following normalization is applied after each step in the core.

Then, another multi-head attention module takes the scene to apply cross-attention between the nucleotide sequences. This component is at the very core of the model because it extracts binding relations between RNA sequences. Here, one sequence is given to the Query input, and the other sequence goes to Value and Key inputs, which is compatible with the original transformer design [26].

Finally, a dense projection component applies two dense layers for extracting meaningful binding information from data coming from multi-head attention components.

### Final components

At the final stages of the model, we have some common components widely adopted in neural networks.

A pooling module performs dimensionality reduction and makes the model more resistant to small changes in the input.

A dropout component is used to prevent overfitting and improve generalization.

At the output layer, three dense components extract the final binding information out of the model.

## Experimental setup

The beginning code for the general transformer models is taken from the book titled “Deep Learning with Python” by François Chollet [27]. Then, the code is modified to implement the proposed rbpTransformer, the complete deep-learning model, and other design alternatives discussed in the following section. The proposed AI model has been implemented in Python 3.10 and has run in Google COLAB using A100 GPU devices. The provided system RAM is 83.5 GB, and the GPU RAM is 40.0 GB. Python libraries Tensorflow, Keras, Scikit-learn, Pandas, NumPy, and Matplotlib have been used to implement some parts of the model.

The piRNA and mRNA dataset is taken from online resources of the paper by Yang and colleagues [3]. The datasets are summarized in Table 2. 20% of the datasets are spared for testing, 25% of the remaining data are spared for validation during training, and the rest are used for training. WT_CLASH dataset is used to refine the model empirically since it has many more samples and is more suitable for deep learning. Both WT_CLASH and CSR-1_CLASH datasets have been employed for cross-validation and testing the accuracy of the final model. WT_CLASH dataset is split into two files, positive and negative. In the positive data file, 60438 piRNA and mRNA pairs are listed, known to bind to each other. Similarly, the negative data file lists 60438 RNA pairs, known as non-binding.

**Table 2:** Datasets used in the experiment [3].

Dataset	Number of Positive Samples	Number of Negative Samples
**WT_CLASH**	60438	60438
**CSR-1_CLASH**	10000	10000

WT_CLASH is suitable for deep learning with its large size. CSR-1_CLASH has insufficient samples for deep learning, but it will be used for a complete evaluation.

## Empirical refinement of the model

Several design alternatives need to be evaluated before deciding on the final arrangement of the AI model.

### Optimization algorithm

The first decision that we need to make is about the optimization algorithm. For interested readers, Ruder provides a good summary of these widely adopted optimizers [28]. Keras library provides the most used solutions in practice; they are also employed in this study.

**Fig 2:**
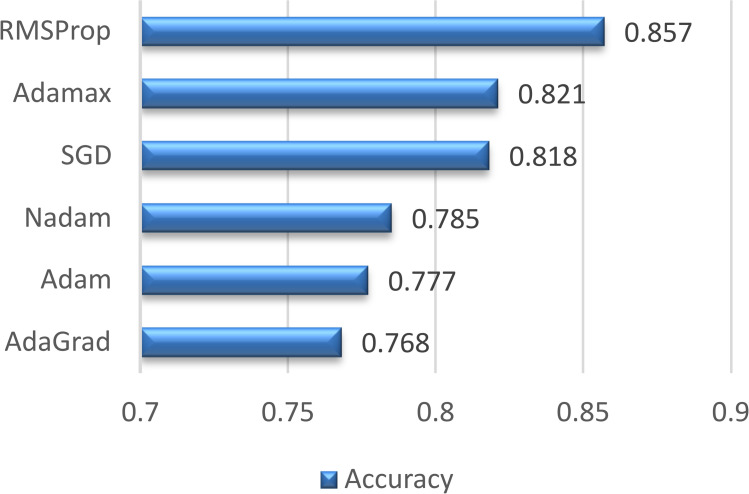
Accuracy values of various optimization algorithms for rbpTransformer model. For choosing the optimizer, 1 core is used, maximum k-mer size is taken as 3.

Adam [29] is usually the best performer in many cases, but here we see a surprising result: it is the second worst with 0.777 accuracy. Other test dataset accuracy values are 0.768 for Adagrad [30], 0.785 for Nadam [31], 0.818 for SGD, 0.821 for Adamax, and 0.857 for RMSProp [32]. Fig 2 depicts the results. Since RMSProp performs much better than other optimizers, this algorithm will be used for the rest of the experiments.

### Impact of k-mer size

K-mers are the main blocks of data in the rbpTransformer AI model. Finding the optimal k-mer size is important to get the best accuracy values and resource usage metrics. Too small or too large k-mer sizes usually result in worse accuracy values, and large k-mer sizes lead to high memory usage and long training times.

**Fig 3:**
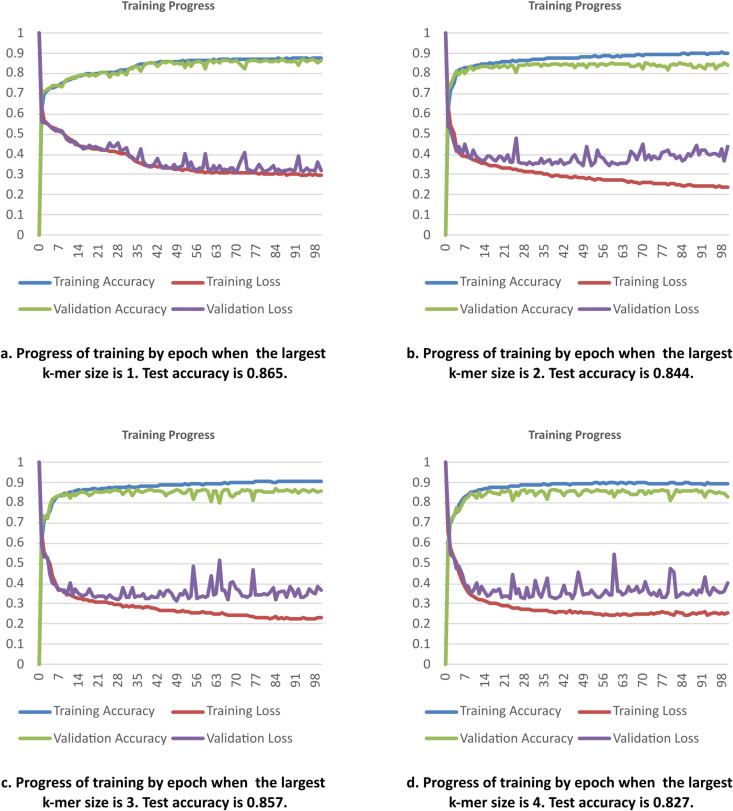
Training Progress for changing k-mer sizes. Training and validation results get separated as we increase the k-mer size, and larger fluctuations are observed for larger k-mer sizes.

Fig 3 shows the training process of four different situations as we change k from 1 to 4. When k is 1, the validation accuracy is closest to the training accuracy, which means overfitting is at the lowest level. The final test accuracy value is equal to 0.865.

When we use maximum k-mer sizes of 2, 3, or 4, the deviation between training and validation lines increases. As the k-mer size increases, the fluctuations in the validation loss get stronger, which can be interpreted as increasing overfitting. Table 3 shows the results of the testing dataset for changing k-mer sizes. When the model was trained with k-mer sizes up to 5, accuracy on the training dataset reached 0.9897 (not shown in the table), but test accuracy fell to 0.782. This is a clear overfitting indication. So, in this subsection, we may conclude that searching the best model for k-mer sizes 1, 2, and 3 would be reasonable since overfitting causes accuracy loss after k-mer size 4.

**Table 3:** Test results for changing k-mer sizes.

Max k-mer size	Precision	Recall	F1 Score	Accuracy
**1**	0.898	0.824	0.859	0.865
**2**	0.824	0.873	0.848	0.844
**3**	0.870	0.838	0.854	0.857
**4**	0.921	0.714	0.804	0.827
**5**	0.770	0.802	0.786	0.782

### With or without self-attention

**Fig 4:**
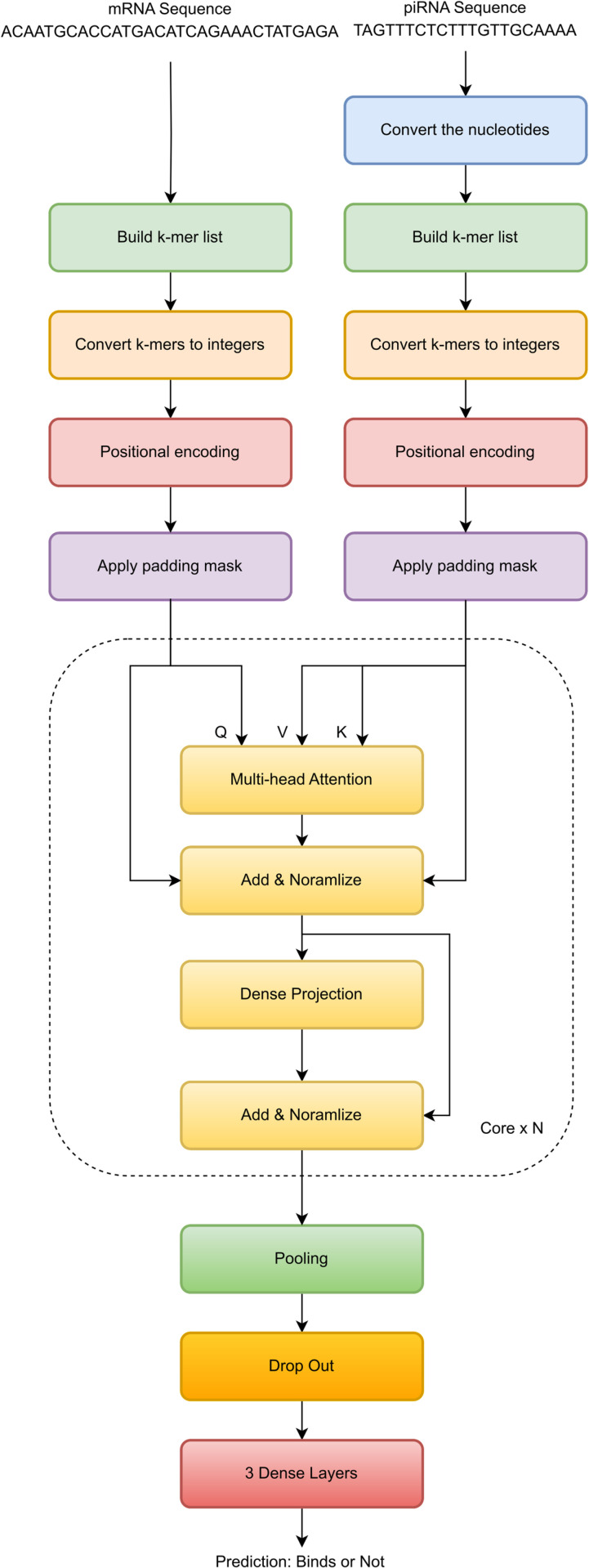
rbpTransformer without self-attention. This model only focuses on cross-sequence relationships.

The self-attention mechanism is at the heart of natural language processing transformers. They must extract information about the meaning of words in a sentence, and the self-attention module reveals the inner relation of words. However, someone can argue that in the RNA binding prediction problem, the self-attention module may worsen the performance since the main concern is finding the binding patterns in two sequences. To evaluate the accuracy of a model using only cross-sequence attention, the deep-learning model shown in Fig 4 is designed and implemented. The self-attention modules have been removed, and inputs are directly fed into a multi-head attention component that takes inputs from the mRNA sequence and the piRNA sequence.

Table 4 shows the results of the test dataset. The model with self-attention performs slightly better with accuracy values of 0.857 to 0.849. This might be due to inferring binding relations concerning the inner orientation of k-mers in a sequence. So, in the rest of the refinement steps, self-attention will be used together with cross-attention.

**Table 4:** Test results comparing versions with and without self-attention.

Self-Attention	Precision	Recall	F1 Score	Accuracy
**Yes**	0.870	0.838	0.854	0.857
**No**	0.847	0.858	0.852	0.849

### Backward k-mers

The next consideration is whether to add backward k-mers to the k-mer list. We must evaluate their impact because critical subsequences in backward order might affect the binding.

For example, let us take a short imaginary nucleotide sequence such as ATGCA. Let us take k as 3; the algorithm will form the following forward k-mers:

A, T, G, C, A, AT, TG, GC, CA, ATG, TGC, GCA

If we also want to include the backward k-mers, the list will be formed as shown below. (Backward k-mers are underlined.)

A, T, G, C, A, AT, TA, TG, GT, GC, CG, CA, AC, ATG, GTA, TGC, CGT, GCA, ACG

So, each k-mer that is longer than one nucleotide is added to the list, reversed, and the backward k-mer is added.

Table 5 shows the test results comparing two models that work with and without backward k-mers. The model with backward k-mers gives an accuracy of 0.816, much less than the one with only forward k-mers. So, we will do our remaining refining with the model that works with forward k-mers only.

**Table 5:** Test results comparing versions with and without backward k-mers.

Backward K-mers	Precision	Recall	Accuracy	F1 Score
**Yes**	0.870	0.838	0.857	0.854
**No**	0.885	0.722	0.816	0.795

### Number of core modules

Finally, we need to decide on the optimal core number. One core is the collection of yellow components in the middle of the deep AI model in Fig. 1. A core includes two multi-head attention modules for self-attention, one multi-head attention module for cross-attention, one dense projection module and four add & normalize modules used after each step.

**Fig 5:**
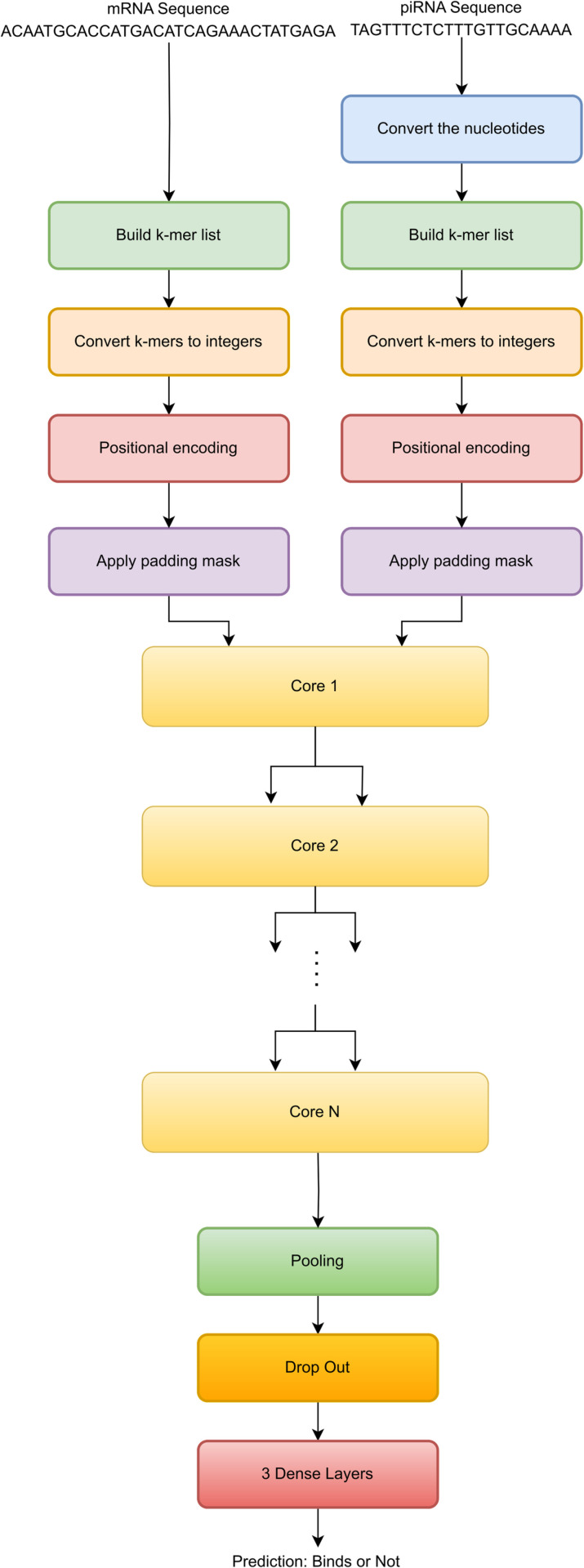
rbpTransformer model using multiple cores. The core component in Fig 1, marked with yellow, is repeated N times.

These core modules can be cascaded one after another to improve the learning status. A new layer will take partially learned data from the previous layer and further perform learning, as shown in Fig 5.

However, after some point, the model will need to train too many parameters, which will become very difficult, and we may expect poor results because the optimal parameter values diverge.

**Fig 6:**
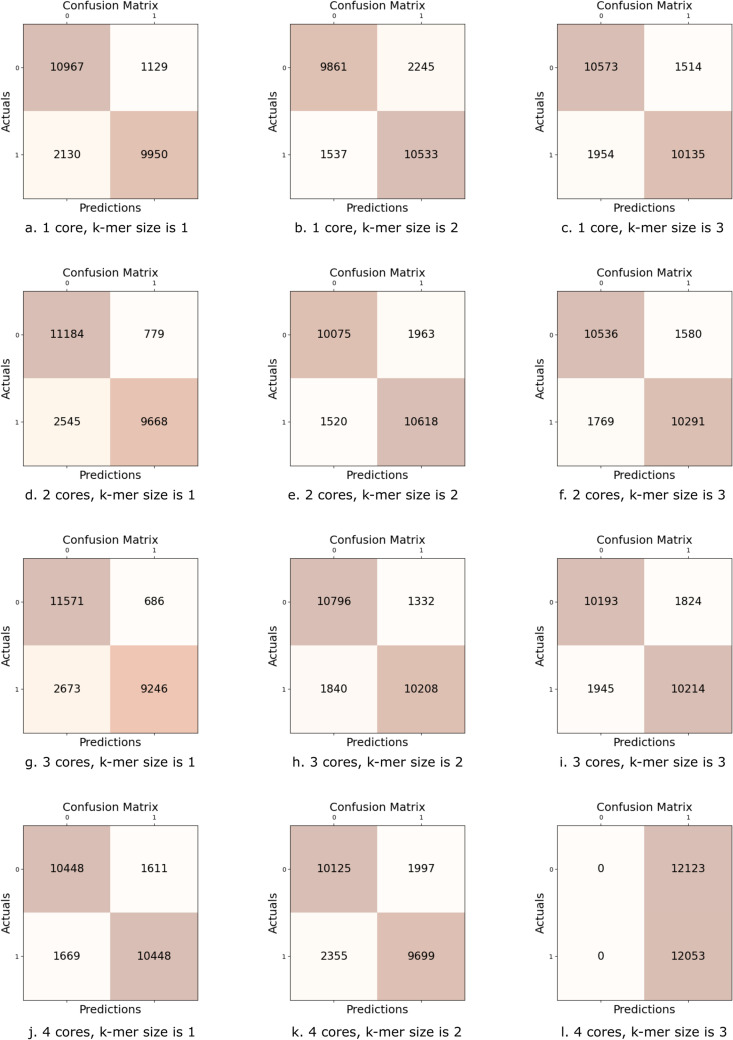
Confusion matrices for varying core numbers and k-mer sizes. Figs 6c, 6e, 6f, 6h, 6i, and 6j are well-balanced, showing over 10,000 correct predictions for positive and negative cases. In Fig 6l, a clear deviation from the correct operation is evident.

Fig 6 depicts the results of 12 experiments changing the core number from 1 to 4 and, for each core number, changing the maximum k-mer size from 1 to 3. We wish the model to behave in a balanced manner so we can look for results with true predictions for both positive and negative cases. Figs 6c, 6e, 6f, 6h, 6i, and 6j are quite balanced. They all show more than 10,000 correct predictions for both positive and negative cases. In Fig 6l, we see a clear divergence from the correct operation. The model predicts all the test cases as positive, which is undesirable. Due to the high number of parameters, the model cannot train the parameters. It is important to note that this case differs from overfitting because the training accuracy always remains around 0.5, which tells us the model cannot learn from the training dataset.

Table 6 depicts the results obtained from test datasets. The number of parameters to train, total training time, accuracy, F1 score, precision, and recall values are shown for each configuration in this table. The most important metric for our problem is the accuracy value. The best accuracy value, 0.869, is obtained using three cores with the maximum k-mer size of 2. So, our final model is determined. Now, we can evaluate our model on two different datasets using cross-validation.

**Table 6:** Test results for changing core numbers and k-mer sizes.

Core Number	Maximum k-mer Size	Number of Parameters to Train	Total Training Time (sec)	Precision	Recall	F1 Score	Accuracy
**1**	**1**	36418	406	0.898	0.824	0.859	0.865
**1**	**2**	119820	1126	0.824	0.873	0.848	0.844
**1**	**3**	258911	1514	0.870	0.838	0.854	0.857
**2**	**1**	62303	1322	0.925	0.792	0.853	0.853
**2**	**2**	218335	1545	0.844	0.875	0.859	0.856
**2**	**3**	472121	1960	0.867	0.853	0.860	0.861
**3**	**1**	88188	1640	0.931	0.776	0.846	0.861
**3**	**2**	316850	1926	0.885	0.847	0.866	0.869
**3**	**3**	685331	2344	0.848	0.840	0.844	0.844
**4**	**1**	114073	2107	0.866	0.862	0.864	0.864
**4**	**2**	415365	2289	0.829	0.805	0.817	0.820
**4**	**3**	898541	2489	0.499	1.000	0.665	0.499

## Experimental results

### Tests on WT_CLASH dataset

The model is trained on different samples from the dataset [3] 10 times. Each time, the dataset is shuffled randomly, and different samples are placed in training, validation, and test datasets. Table 7 shows the results. After 10 runs on randomly determined training and test datasets, the average accuracy is 0.859.

**Table 7:** Cross-validation on the WT_CLASH dataset.

Run	Precision	Recall	F1 Score	Accuracy
**1**	0.885	0.847	0.866	0.869
**2**	0.953	0.721	0.821	0.844
**3**	0.893	0.823	0.857	0.864
**4**	0.880	0.870	0.875	0.876
**5**	0.889	0.870	0.879	0.880
**6**	0.889	0.770	0.825	0.838
**7**	0.841	0.878	0.859	0.857
**8**	0.792	0.915	0.849	0.837
**9**	0.890	0.853	0.871	0.874
**10**	0.821	0.906	0.861	0.855
**Average**	0.873	0.845	0.856	0.859

### Tests on CR-1_CLASH dataset

The accuracy results are quite acceptable for the WT_CRASH dataset, which has 120,876 samples. But how about datasets with fewer samples?

It is well known that deep learning can only work on large datasets. This is because we cannot train a large number of parameters with a small number of samples. To be complete, we must show the results on a small dataset. We will use the CR-1_CRASH dataset [3], which has 20,000 samples in total. The results are shown in Table 8. The average accuracy is 0.721, much less than the value obtained from the other dataset. The accuracy on the training dataset has surpassed 0.95 during some runs, so we can say that the model overfits itself to training data and cannot be so accurate on test data due to insufficient training.

**Table 8:** Cross-validation on the CR-1_CLASH dataset.

Run	Precision	Recall	F1 Score	Accuracy
**1**	0.721	0.631	0.673	0.691
**2**	0.725	0.712	0.719	0.722
**3**	0.749	0.710	0.729	0.736
**4**	0.724	0.759	0.741	0.731
**5**	0.766	0.695	0.729	0.740
**6**	0.700	0.786	0.740	0.723
**7**	0.712	0.722	0.717	0.711
**8**	0.763	0.646	0.700	0.722
**9**	0.719	0.717	0.718	0.721
**10**	0.694	0.756	0.724	0.715
**Average**	0.727	0.713	0.719	0.721

### Tests targeting AUC

The dataset provider of this research reports the results mostly using AUC (Area Under Curve) metric. Actually, AUC can be regarded as a better metric then accuracy, because it includes evaluation of possible threshold values and gives the success of the model independent of chosen threshold.

So, additional training has been performed targeting high AUC. The results are listed in Table 9. The average AUC after 10 runs on randomly determined training, validation and test datasets is 94.38%, as far as we know, this is the best AUC value obtained on this dataset so far.

**Table 9:** Cross-validation on the WT_CLASH dataset targeting AUC.

Run	AUC
**1**	0.955
**2**	0.938
**3**	0.946
**4**	0.937
**5**	0.938
**6**	0.944
**7**	0.950
**8**	0.949
**9**	0.943
**10**	0.938
**Average**	0.9438

### Comparison with previous models

**Fig 7:**
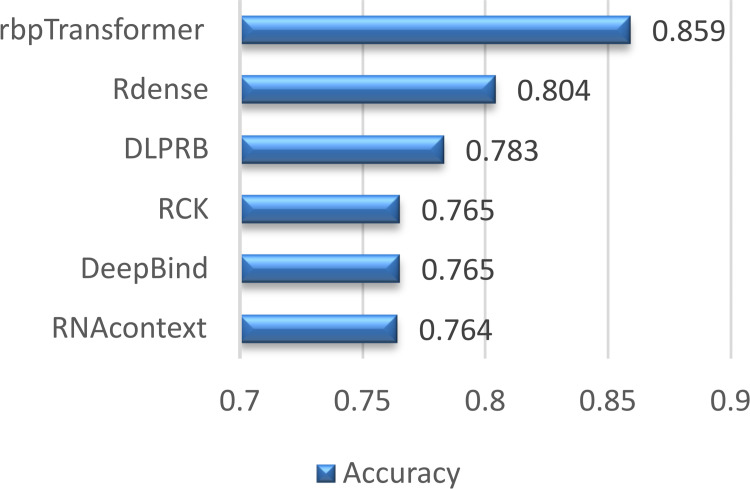
Accuracy values of rbpTransformer and some outstanding models in the literature. (Rdense [25], DLPRB [33], RCK [34], RNAcontext [35], DeepBind [15]).

It is not very easy to make a comparison with the models in the literature due to the varying datasets used in different studies, the availability of the training and testing code, and the availability of datasets used. rbpTransformer with some outstanding models in the literature. Fig 7 and Table 10 compare rbpTransformer’s accuracy results on the large-size dataset with some of the models that are mentioned in the paper by Li and colleagues [25]. Fig 7 shows that the rbpTransformer model can be a good candidate for predicting the bindings between piRNAs and mRNAs with an average accuracy of 0.859 when the dataset is large enough to train the model.

**Table 10:** Accuracy values of rbpTransformer and some outstanding models in the literature. (Rdense [25], DLPRB [33], RCK [34], RNAcontext [35], DeepBind [15]).

Model	Accuracy
**rbpTransformer**	0.859
**Rdense**	0.804
**DLPRB**	0.783
**RCK**	0.765
**DeepBind**	0.765
**RNAcontext**	0.764

Finally, Table 11 gives a direct comparison between rbpTransformer and the Multi-head Attention based model of Yang and colleagues [3] that used the same datasets. Rbp Transformer offers a better AUC value showing its potential for being a solution to the important RNA binding problem.

**Table 11:** AUC Comparison.

Model	AUC (%)
**rbpTransformer**	94.38
**Multi-head Attention based model of Yang and colleagues [**3]	93.30

## Discussion

The rbpTransformer deep learning model has been suggested and experimentally validated with a number of design variations. However, the transformation of this computational solution into a reliable tool for different research settings also has certain challenges. The subsequent are the major advantages, major limitations, possible interpretability strategies, and promising extensions for potential research:

### Major advantages

Cross-Attention Advantage: Cross-attention mechanism is particularly designed to capture inter-sequence relationships, which is necessary to identify piRNA–mRNA binding sites.

Self-Attention Integration: By self-attention integration, intrasequence relationships and positional information are discovered, thus overcoming the complex interactions difficult to model with less complex algorithms.

Flexible Design Components: rbpTransfprmer allows for k-mer size tuning and core module number tuning in an effort to align model capacity and reduce overfitting—both typical pitfalls with RNA-binding prediction.

### Limitations and dataset constraints

Although rbpTransformer achieves high accuracy on sufficiently large datasets, smaller ones (e.g., CSR-1_CLASH) result in overfitting and reduced performance. This indicates a dataset dependence, as deep models typically require large training datasets. Possible future work involves semi-supervised learning or data augmentation (e.g., reverse-complement sequences) to alleviate this vulnerability.

### Computational overheads

Training a transformer can be computationally expensive, requiring large memory and GPU capacities. Approximate attention or model compression techniques can mitigate hardware requirements, making rbpTransformer more accessible to laboratories with lower-performance hardware.

### Interpretability

As mentioned for most deep learning methods, rbpTransformer is also a “black box.” The effectiveness of attention heatmaps or saliency maps may be used to unveil what subsequence features contribute to binding predictions. SHAP values may also offer quantitative information about the contribution of every single base (or k-mer) to ultimate results.

### Multi-omics integration

Although the proposed model herein is focused on sequence-based features, incorporating multi-omics data (e.g., RNA expression, chromatin accessibility, protein interactions) can further improve prediction quality. One potential pipeline can combine those data sources with the cross-attention mechanism to include interactions beyond primary sequence signals.

### Wet-lab validation

Computational predictions are greatly benefited by validation using experimental assays such as CLIP-seq, RIP-seq, or luciferase tests. Partnerships with experimental labs can refine the outputs of rbpTransformer, bridging the gap between in silico models and in vitro or in vivo experiments.

## Conclusion

A novel deep-learning model for predicting piRNA and mRNA bindings and its empirical refinement has been introduced. The model is a good candidate for large enough datasets. The model uses both self-attention and cross-attention in the core component. The core component can be cascaded several times to improve the model’s accuracy. Empirical refinement showed that the RMSProp optimizer is more suitable for the model and dataset used in this research. Using self-attention with cross-attention improves the accuracy results. Backward k-mers do not improve performance; on the contrary, they lead to lower accuracy values. Grid search on the core number and the largest k-mer size showed that using 2 cores and k-mers with size 3 at most gives the best accuracy values. Cross-validation on two datasets and comparison with outstanding models in the literature shows the model can be a good solution if enough samples are provided. Future work can focus upon interpretability features, optimize resource usage, and verify predictions via wet-lab experiments, thus ensuring rbpTransformer’s broader applicability in piRNA–mRNA interaction research.
